# Spatial variability of hosts, parasitoids and their interactions across a homogeneous landscape

**DOI:** 10.1002/ece3.6158

**Published:** 2020-03-04

**Authors:** Anna Torné‐Noguera, Xavier Arnan, Anselm Rodrigo, Jordi Bosch

**Affiliations:** ^1^ CREAF Cerdanyola del Vallès Spain; ^2^ Universitat Autònoma de Barcelona Cerdanyola del Vallès Spain

**Keywords:** beta‐diversity, homogeneous habitat, host‐parasitoid food web, local scale, spatial variation, species interactions, trap‐nests

## Abstract

Species assemblages and their interactions vary through space, generating diversity patterns at different spatial scales. Here, we study the local‐scale spatial variation of a cavity‐nesting bee and wasp community (hosts), their nest associates (parasitoids), and the resulting antagonistic network over a continuous and homogeneous habitat. To obtain bee/wasp nests, we placed trap‐nests at 25 sites over a 32 km^2^ area. We obtained 1,541 nests (4,954 cells) belonging to 40 host species and containing 27 parasitoid species. The most abundant host species tended to have higher parasitism rate. Community composition dissimilarity was relatively high for both hosts and parasitoids, and the main component of this variability was species turnover, with a very minor contribution of ordered species loss (nestedness). That is, local species richness tended to be similar across the study area and community composition tended to differ between sites. Interestingly, the spatial matching between host and parasitoid composition was low. Host β‐diversity was weakly (positively) but significantly related to geographic distance. On the other hand, parasitoid and host‐parasitoid interaction β‐diversities were not significantly related to geographic distance. Interaction β‐diversity was even higher than host and parasitoid β‐diversity, and mostly due to species turnover. Interaction rewiring between plots and between local webs and the regional metaweb was very low. In sum, species composition was rather idiosyncratic to each site causing a relevant mismatch between hosts and parasitoid composition. However, pairs of host and parasitoid species tended to interact similarly wherever they co‐occurred. Our results additionally show that interaction β‐diversity is better explained by parasitoid than by host β‐diversity. We discuss the importance of identifying the sources of variation to understand the drivers of the observed heterogeneity.

## Graphical abstract

We demonstrate that communities of mobile organisms and their interactions vary at a local scale (~500 m) even in a continuous and homogeneous habitat. Parasitoid, rather than host, spatial turnover is the major driver of the observed spatial heterogeneity in host‐parasitoid interactions. Nonetheless, interactions are also subjected to their own intrinsic variability.

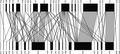

## 1 INTRODUCTION

Diversity patterns we observe in nature are the outcome of multiple biotic and abiotic factors and the interactions occurring among them. Understanding these patterns and their underlying processes is one of the main goals of community ecology (Gaston, 1996). For many decades, ecologists have studied species diversity patterns at scales ranging from several meters to thousands of kilometers. Whittaker (1960) was the first to propose a partitioning of diversity across three different spatial scales: α‐diversity, that is, species richness in a particular locality, γ‐diversity, a measure of regional species richness, and β‐diversity, a measure of species composition dissimilarity between localities. β‐diversity provides a measure of community spatial variability, reflecting historical processes, and revealing information on population dynamics and species responses to habitat modifications such as environmental gradients and perturbations (Cavender‐Bares, Kozak, Fine, & Kembel, 2009; Graham & Fine, 2008). For this reason, β‐diversity has become a relevant measure in biological conservation (Condit et al., 2002). β‐diversity can be partitioned into two additive components: species turnover (dissimilarity due to species replacement), and nestedness of assemblages (dissimilarity due to ordered species loss; Baselga, 2010). Knowing the relative importance of these two components is essential to understand the causes of observed spatial variability.

Because species are not isolated but immersed in complex networks connecting them directly and indirectly with other species, spatial heterogeneity in species community structure is expected to profoundly affect interaction network structure (Olesen & Jordano, 2002; Vázquez, Blüthgen, Cagnolo, & Chacoff, 2009). An increasing number of studies are either theoretically or empirically addressing such relationship (Burkle & Alarcón, 2011). For example, spatial aggregation, plant identity, and animal mobility have a strong influence on interaction identity, strength, and distribution in plant–animal mutualistic networks (Morales & Vázquez, 2008). Others show that network structural patterns are largely shaped by relative species abundance and spatiotemporal patterns of interacting species (CaraDonna et al., 2017; Trøjelsgaard, Jordano, Carstensen, & Olesen, 2015; Vázquez, Chacoff, & Cagnolo, 2009).

The decay of similarity in species composition with geographical distance is well established (Carstensen, Sabatino, Trøjelsgaard, & Morellato, 2014; Dáttilo, Guimarães, & Izzo, 2013; Trøjelsgaard et al., 2015), and usually explained as a result of decreasing environmental similarity and/or dispersal limitation (Nekola & White, 1999; Soininen, McDonald, & Hillebrand, 2007). However, little is known about how interaction similarity decays and how this decay is related to species similarity decay. Because interactions are influenced by the variability of the two trophic levels plus their inherent variability (i.e., same species interacting differently), interactions are expected to display more spatial variation than species (Poisot, Guéveneux‐Julien, Fortin, Gravel, & Legendre, 2015; Poisot, Stouffer, & Gravel, 2017). Although β‐diversity is widely used among ecologists to explore community spatial variation, it has been seldom used to explore the spatial variation of interactions. Poisot, Canard, Mouillot, Mouquet, and Gravel (2012) proposed a dissimilarity index to explore differences between interaction networks across space. To additionally delve into the source of variability between networks, they subdivided β‐diversity of interactions into two components. The first component includes dissimilarity due to species turnover and the second dissimilarity due to interaction turnover (same species interacting differently, or rewiring). Poisot et al. (2012) also proposed the comparison of interactions of each local web to its counterpart in the regional web as a means to determine whether interactions found at the regional level are always found at the local level (which would indicate that species interact similarly across the region). This approach provides a measure of the extent to which realized and potential interactions differ, that is, the intensity of interaction filtering.

Recently, some studies have empirically addressed β‐diversity of mutualistic (CaraDonna et al., 2017; Carstensen et al., 2014; Costa et al., 2018; Dáttilo et al., 2013; Dáttilo & Vasconcelos, 2019; Luna, Peñaloza‐Arellanes, Castillo‐Meza, García‐Chávez, & Dáttilo, 2018; Norfolk, Eichhorn, & Gilbert, 2015; Simanonok & Burkle, 2014; Trøjelsgaard et al., 2015) and antagonistic (Novotny, 2009; Poisot et al., 2012, 2017) interactions. However, our understanding of the mechanisms underlying the distribution of interactions is still fragmentary. For instance, there is no consensus on the relative contribution of species turnover and rewiring to interaction dissimilarity. Current evidence suggests that species turnover is more important than rewiring across spatial gradients (Novotny, 2009, Poisot et al., 2012, Carstensen et al., 2014, Trøjelsgaard et al., 2015; but see Dáttilo & Vasconcelos, 2019), whereas rewiring is more important across temporal gradients (Caradonna et al., 2017; Costa et al., 2018; Luna et al., 2018). It is also important to understand how the species turnover of each trophic level contributes to interaction turnover. This could help find proxies of species interactions and thus improve sampling efficiency. So far, most studies have found turnover of the lower trophic level to be the major contributor of interaction turnover (Carstensen et al., 2014; Norfolk et al., 2015; Novotny, 2009; Simanonok & Burkle, 2014). However, such relationships may be blurred by methodological artifacts and at least one study attributes a greater role to the higher trophic level (Trøjelsgaard et al., 2015).

Here, we work with a community of cavity‐nesting bees and wasps (henceforth hosts) and their nest associates, and study the spatial variation of the two communities and of their interactions. Nest associates include species from different arthropod groups (coleopterans, dipterans, hymenopterans, mites) and with different parasitic habits (parasitoids, cleptoparasites, predators, and scavengers). Because they perform a similar ecosystem function by contributing to the mortality of the host offspring, nest associates are usually treated as a single group in the cavity‐nesting bee/wasp literature, and are referred to as “parasitoids” for convenience (see Staab, Pufal, Tscharntke, & Klein, 2018 for a review). Host‐parasitoid systems are suitable models in which to analyze spatial variability in species composition and to test the ecological mechanisms accounting for spatial variation in interactions. First, parasitoids develop on or within the body of their hosts and therefore are intimately linked to them and experience similar microenvironmental conditions during their development. This is especially true for nest associates, which develop within the nest of their hosts. For this reason, it is usually assumed that host‐parasitoid interactions are more specialized than other kinds of interactions such as predator–prey (Price, 1997; Schowalter, 1996) and mutualistic (excluding symbionts) (Blüthgen, Menzel, Hovestadt, Fiala, & Blüthgen, 2007; Fontaine, Thébault, & Dajoz, 2009). Therefore, we should expect a strong match between the distribution of parasitoids and that of their hosts (Poisot et al., 2017; Poulin, Krasnov, & Mouillot, 2011).

Previous studies working with cavity‐nesting bees and wasps analyze composition differences between seasons, habitats, or environmental gradients (Albrecht, Duelli, Schmid, & Müller, 2007, Tylianakis, Tscharntke, & Lewis, 2007, Osorio, Arnan, Bassols, Vicens, & Bosch, 2015, Osorio‐Canadas et al., 2018, Morris et al., 2014, Staab et al., 2016; review in Staab et al., 2018). Conversely, our study was conducted across a habitat that can be considered uniform (at a landscape scale), with no physical barriers or strong environmental gradients and dominated by a single vegetation type, and addresses spatial variability at a local scale (distance between plots is ca. 1,000 m). Therefore, our aim is to study the intrinsic variability of the host‐parasitoid network and its underlying mechanisms, rather than to establish how different environmental factors may affect interaction identity and network structure.

We have the following objectives: (a) To analyze the spatial variation of the host and parasitoid communities across a continuous habitat; (b) To measure the β‐diversity of host‐parasitoid interactions; (c) To test whether species interact similarly across the study region; and (d) To analyze the relationship between host, parasitoid and interaction β‐diversity. We developed four hypotheses. First, because our study organisms are highly mobile (Gathmann & Tscharntke, 2002, Klein, Steffan‐Dewenter, & Tscharntke, 2004, Greenleaf, Williams, Winfree, & Kremen, 2007, Guedot et al., 2009, Cronin & Reeve, 2005), we should expect low spatial variation in community composition. Yet, recent studies in the same study area reported high levels of spatial heterogeneity in pollinator (Reverté et al., 2019) and bee species composition (Torné‐Noguera et al., 2014), despite similar levels of abundance and richness. We, thus, hypothesize (H1) moderate to high spatial variation in species composition for both taxa, and we expect this variation to be mainly due to species turnover (as opposed to nestedness). Second, because parasitoids necessarily depend on their hosts and usually have relatively narrow host ranges (Hawkins, 1994; Jeffs & Lewis, 2013; Poulin et al., 2011), we hypothesize (H2) a strong match in species composition across space and similar distance decay patterns for both groups. Third, we expect higher heterogeneity in interactions than in species composition (Poisot et al., 2017,2015), and because we work in a spatial (as opposed to temporal) gradient, we hypothesize (H3) a higher contribution of species turnover than rewiring to overall interaction dissimilarity. Assuming that host‐parasitoid associations are relatively narrow, we expect a similar distance decay pattern for interaction similarity than for host and parasitoid similarity. For the same reason, we also expect low levels of interaction filtering (species interacting similarly across the study region). Fourth, interaction turnover is often dependent on variation of the lower trophic level (Carstensen et al., 2014; Norfolk et al., 2015; Novotny, 2009; Simanonok & Burkle, 2014), thus we hypothesize (H4) that interaction β‐diversity will be better predicted by host β‐diversity than by parasitoid β‐diversity.

## 2 MATERIAL AND METHODS

### 2.1 Study area

The study was conducted in the Garraf Natural Park (Barcelona, NE Spain), a Mediterranean scrubland dominated by *Quercus coccifera*, *Pistacia lentiscus*, *Rosmarinus officinalis* and *Thymus vulgaris*. Our study area encompasses a surface of 32 km^2^. We selected 25 plots distributed across the study area (Appendix S1). Distances between nearest plots ranged from 585 to 1,354 m. As mentioned, the study area is homogeneous at a landscape scale, being occupied by a single type of vegetation with similar physiognomic features (Reverté et al., 2019; Torné‐Noguera et al., 2014).

### 2.2 Surveys

In each plot, we placed a trap‐nesting station facing *SE*. Each station contained seven drilled wood blocks with inserted paper tubes. Each wood block accommodated 25 tubes of one of the following diameters (2, 3, 4, 5, 6, 7, and 8 mm), resulting in 175 nesting cavities per station. Paper tube length was 5 cm for the 2 and 3 mm diameters and 15 cm for the rest. Nesting stations were checked every 2 weeks and tubes containing occupied nests were pulled out, taken to the laboratory and replaced with empty ones, so that there were nesting cavities of all diameters available at all times. Nesting stations remained in the field from February to October, in 2011 and 2013. Data of the two years are pooled together in the analyses.

Nests were kept in a temperature chamber simulating monthly ambient temperatures of the study site. Upon one year from collection, nests were dissected. The nests of solitary bees and wasps contain a variable number of cells, some of which may be parasitized. We recorded the contents of each cell (identity of the host or the parasitoid).

### 2.3 Statistical analysis

Sampling completeness was assessed by building a mean sample‐based host species accumulation curve from 1,000 curves based on random sample additions and calculating the Ace Mean species richness estimator (Estimates 8.0.0; Colwell, 2006). We did not build accumulation curves for parasitoids because our sampling approach is host‐centered and parasitoids can only be detected if they have parasitized a host.

#### 2.3.1 Host and parasitoid communities

To characterize community structure at each nesting station, we used the variables host abundance (number of host cells produced, including both non‐parasitized and parasitized cells), host richness (number of host species), parasitoid abundance (number of cells parasitized), parasitoid richness (number of parasitoid species), and parasitism rate (% cells parasitized).

We measured the correlation (Spearman's rho) between host abundance and richness (for all hosts and for parasitized hosts separately), between parasitoid abundance and richness, and between host (all hosts) and parasitoid abundance and richness.

To analyze the spatial variation in host and parasitoid species composition and the relative contribution of species turnover and species nestedness to this variation, we used the species β‐diversity partition framework proposed by Baselga (2010). To obtain a measure of diversity for each site‐to‐site comparison, we calculated β‐diversity based on Sørensen's dissimilarity index for the host (β_H_), parasitized host (β_PH_), and parasitoid (β_P_) communities. This index accounts for the total compositional variation between assemblages, including both turnover and nestedness components. β‐diversity was then broken down into the component due to species turnover (β_SIM_) (species replacement), and the component due to species nestedness (β_SNE_) (species loss; Baselga, 2010). β_SIM_ is the Simpson dissimilarity index and is invariant to richness differences. β_SNE_ is calculated as the difference between the Sørensen and Simpson indices. These pairwise metrics use information on the number of species shared between two sites and the number of species unique to each site. These analyses were conducted with the *betapart* package (Baselga & Orme, 2012) for R (R Core Team, 2014).

Correlations between host β‐diversity (all hosts (β_H_) and parasitized hosts (β_PH_) separately) and parasitoid β‐diversity (β_P_) were tested with Mantel tests based on Spearman's rho. To analyze distance decay of the different components of β‐diversity, we again used Mantel tests to calculate correlations between geographical distance and host β‐diversity (β_H_), parasitized host β‐diversity (β_PH_), and parasitoid β‐diversity (β_P_).

#### 2.3.2 Interactions

We first conducted two general linear models (GLMs) to analyze the relationship between parasitism rate and host abundance at the plot level, and between species abundance and parasitism rate at the species level.

We then built an interaction network for each plot (25 local webs), and a regional network (metaweb) pooling the data from all plots. We explored local web dissimilarity by measuring β‐diversity of interactions among local networks (β_WN_). Following Poisot et al. (2012), this dissimilarity was subdivided into dissimilarity due to species turnover (β_ST_) and dissimilarity due to interaction rewiring (β_OS_). Sometimes, it was not possible to calculate β_OS_ due to the lack of shared species between two plots. In these cases, all network variability (β_WN_) was assigned to species turnover (β_ST_). In addition, and again following Poisot et al. (2012), we calculated β’_OS_, a measure of dissimilarity of interactions between local webs and their counterparts in the regional web. β’_OS_ measures the extent to which the realized and potential interactions differ in each local community (i.e., the intensity of interaction filtering across the region). All these indexes were calculated with *betalink* package for R.

To analyze distance decay of the different components of network β‐diversity, we performed Mantel tests based on Spearman rho between geographical distance and interaction β‐diversity (β_WN_), β‐diversity due to species turnover (β_ST_) and β‐diversity due to rewiring (β_OS_).

We were interested in determining whether interaction β‐diversity (β_WN_) was better predicted by the spatial variability of the host trophic level or that of the parasitoid trophic level. To this aim, we performed two GLMs in which the response variable was interaction β‐diversity (β_WN_) and the explanatory variables were parasitoid β‐diversity (β_P_) and either host β‐diversity (β_H_) or parasitized host β‐diversity (β_PH_). Because explanatory variables could be correlated (see results), we calculated the variance inflation factor (VIF) to make sure collinearity was low (VIF = 1.05 for the all hosts model, and 1.17 for the parasitized hosts model). Geographic distance was not included in these models because our results showed it did not have a strong effect on interaction β‐diversity (see Results). The percentage of variance explained by the model was calculated with *pmvd* metric using *relaimpo* package (Grömping, 2006) for R.

## 3 RESULTS

**FIGURE 1:**
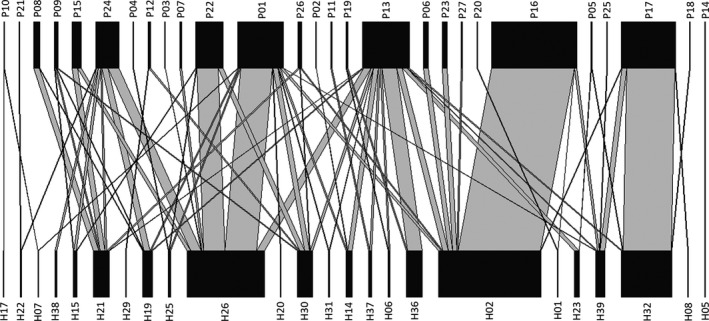
Regional host‐parasitoids web (metaweb of 25 plots). Species names can be found in Appendix S2. Only parasitized host individuals are included

We obtained 1,541 nests (4,954 cells) from 40 host species. Seventeen of these species were bees (Megachilidae—15 sp., Colletidae—2 sp.), and 23 were wasps (Crabronidae—12 sp., Pompilidae—5 sp., Vespidae—4 sp., Sphecidae—1 sp., Ampulicidae—1 sp.). Twenty‐four host species had, at least, one individual parasitized. We found 27 parasitoid species (16 wasps, 6 flies, 3 beetles, 1 bee, and 1 mite) and 654 parasitized cells, so parasitism rate was 13.2%. Of the 27 parasitoid species, 18 were true parasitoids, 7 were cleptoparasites, 1 was a predator, and 1 was a scavenger (Figure 1, Appendix S2). The host species accumulation curve approached asymptoticness (Appendix S3). The Ace Mean estimator predicted a total richness of 42 host species, with 98% sampling completeness.

### 3.1 Host community

As expected, host abundance and richness showed a positive relationship, both when considering all hosts (ρ = 0.59, *p* = .002, *n* = 25 plots) and when considering only parasitized hosts (ρ = 0.57, *p* = .003; *n* = 25 plots). All‐host β‐diversity was moderate to high (β_H,_ mean ± *SD* = 0.57 ± 0.14), and mostly due to species turnover (β_SIM‐H_ = 0.47 ± 0.18, 82.5%), with a poor contribution of nestedness (β_SNE‐H,_ mean ± *SD* = 0.10 ± 0.08, 17.5%). Parasitized host β‐diversity was higher (β_PH,_ mean ± *SD* = 0.68 ± 0.21) than all‐host β‐diversity, again mostly due to species turnover (β_SIM‐PH,_ mean ± *SD* = 0.56 ± 0.30, 82.4%), with a weak contribution of nestedness (β_SNE‐PH,_ mean ± *SD* = 0.12 ± 0.16, 17.6%). Therefore, local host communities showed notable differences in species composition, and these differences were mostly due to species replacement rather than species loss.

### 3.2 Parasitoid community

As expected, parasitoid abundance and richness were positively correlated (ρ = 0.57, *p* = .003). Parasitoid β‐diversity was similar to host β‐diversity (β_P,_ mean ± *SD* = 0.60 ± 0.18), and again mostly attributable to species turnover (β_SIM‐P,_ mean ± *SD* = 0.46 ± 0.26, 76.7%) rather than nestedness (β_SNE‐P,_ mean ± *SD* = 0.14 ± 0.16, 23.3%).

### 3.3 Relationship between host and parasitoid β‐diversity

The parasitoid community was highly dependent on the host community: there was a significant positive correlation between parasitoid and host abundance (all hosts: ρ = 0.62, *p* = .001), and between parasitoid and host richness (all hosts: ρ = 0.75, *p* < .0001). β‐diversity of parasitoids (β_P_) was significantly but weakly correlated to β‐diversity of all hosts (β_H_) (Mantel ρ = 0.19, *p* = .011) and to β‐diversity of parasitized hosts (β_PH_) (Mantel ρ = 0.38, *p* = .001).

### 3.4 Distance decay in host and parasitoid community β‐diversity

**FIGURE 2:**
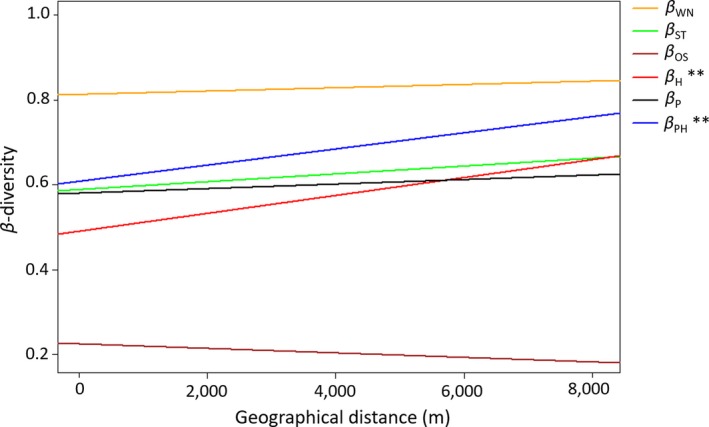
Distance decay of all‐host β‐diversity (β_H_), parasitized host β‐diversity (β_PH_), parasitoid β‐diversity (β_P_), interaction β‐diversity (β_WN_), the component of interaction β‐diversity due to species turnover (β_ST_), and the component of interaction β‐diversity due to rewiring (β_OS_). (Statistical significance; **p ≤ .01)

β‐diversity of all hosts (β_H_) (Mantel ρ = 0.29, *p* = .002) and β‐diversity of parasitized hosts (β_PH_) (Mantel ρ = 0.20, *p* = .004) were significantly but weakly correlated to geographic distance. On the other hand, β‐diversity of parasitoids (β_P_) was not correlated to geographic distance (Mantel ρ = 0.06, *p* = .167) (Figure 2).

### 3.5 Interactions

Parasitism rate was not explained by host abundance at the plot level (GLM: *p* = .75, *n* = 25 plots). However, host species that were more abundant at the regional level had higher parasitism rates (GLM: *p* = .02, pseudo‐R^2^ = 0.13, *n* = 24 species).

We detected 73 specific interactions. Most of these (63.9%) were recorded in just one plot, and 40.3% were recorded only once (i.e., only one individual host was parasitized). Pairwise interaction dissimilarity between plots was high (β_WN_, mean ± *SD*: 0.83 ± 0.16). Most of this dissimilarity was due to species turnover (β_ST_, mean ± *SD*: 0.62 ± 0.32, 74.7%), with a much lesser contribution of interaction rewiring (β_OS_, mean ± *SD*: 0.21 ± 0.31; 25.3%). β’_OS_ values were low (between 0 and 0.31; mean ± *SD*=0.17 ± 0.10). That is, species interacted similarly across local networks, indicating weak interaction filtering across the region.

### 3.6 Distance decay of interaction β‐diversity

As with parasitoid β‐diversity, network β‐diversity (β_WN_: Mantel ρ = 0.08, *p* = .145) was not related to geographical distance. In fact, neither of the two components of network β‐diversity, β‐diversity due to species turnover (β_ST_: Mantel ρ = 0.09, *p* = .109) or β‐diversity due to rewiring (β_OS_: Mantel ρ = −0.03, *p* = .668) (Figure 2) showed significant distance decay.

### 3.7 Relationship between species and interaction β‐diversity

**FIGURE 3:**
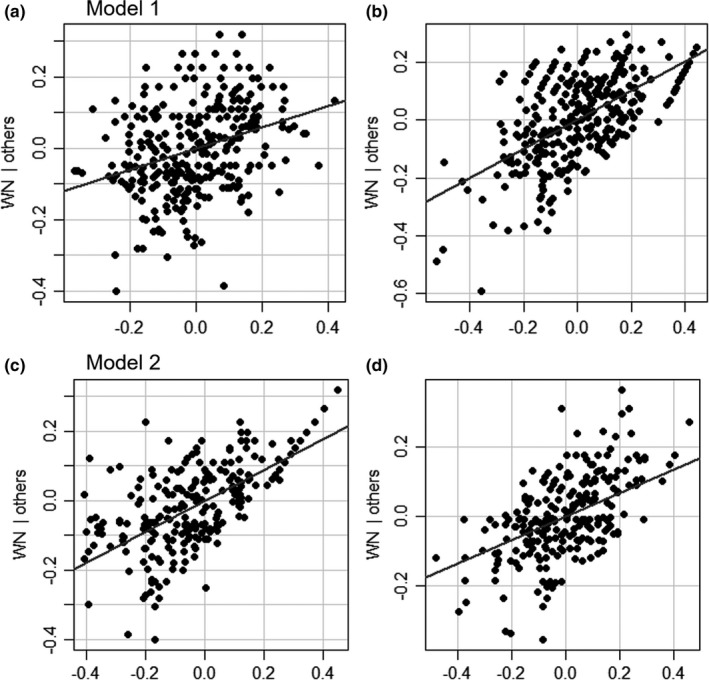
Relationship between all‐host β‐diversity (β_H_), parasitized host β‐diversity (β_PH_), parasitoid β‐diversity (β_P_) (explanatory variables), and interaction β‐diversity (β_WN_) (predicted variable). Model 1 (a, b) includes all hosts (β_H_). Model 2 (c, d) includes only parasitized hosts (β_H_). Partial regression plots obtained from linear models when the effect of the other explanatory variable is removed

All‐host (β_H_), parasitized host (β_PH_) and parasitoid β‐diversity (β_P_) were moderately good predictors of interaction β‐diversity (β_WN_) (Table 1, Figure 3). The model including all hosts explained 43.9% of the variance (33.6% by β_P_ and only 10.3% by β_H_). On the other hand, the model including only parasitized hosts explained as much as 65.6% of the variance (24.4% by β_P_ and 41.2% by β_PH_) (Table 1).

**TABLE 1:** Statistical outputs of GLMs analyzing the contribution of all‐host β‐diversity (β_H_), parasitized host β‐diversity (β_PH_) and parasitoid β‐diversity (β_P_) to interaction β‐diversity (β_WN_). β_H_ (Model 1) includes all hosts; β_PH_ (Model 2) includes only parasitized hosts

	Estimate	*SE*	*t*‐value	*p*	Variance explained (%)
Model 1					37.3
β_H_	0.298	0.049	6.04	<.0001	10.3
β_P_	0.506	0.040	12.6	<.0001	33.6
Model 2					62.8
β_PH_	0.447	0.029	15.7	<.0001	41.2
β_P_	0.338	0.034	10.0	<.0001	24.4

## 4 DISCUSSION

We demonstrate that both our host and parasitoid communities show relatively high spatial variation across a uniform (at the landscape level) habitat. Interestingly, the spatial variation of parasitoids did only weakly match that of hosts. Host dissimilarity increased with geographic distance, whereas parasitoid dissimilarity did not show a distance decay pattern. Host‐parasitoid interactions displayed greater spatial variability than the host and parasitoid communities. Interaction dissimilarity did not show a distance decay pattern and was mostly explained by species turnover with a low contribution of rewiring. In other words, interactions were usually realized whenever the two partners co‐occurred locally, indicating that interaction filtering across space was very low. Finally, we demonstrate that spatial variability in host‐parasitoid interactions is mostly explained by variability of the parasitoid rather than the host community.

Our first objective addressed the spatial variability of the host and parasitoid communities. Although the study was conducted within a uniform habitat, the data that we collected support our first hypothesis that both the host and parasitoid communities showed relatively high spatial variability at a local scale as indicated by the intermediate to high β‐diversity values. Moreover, this high spatial variability was mostly due to species turnover, whereas species loss was almost irrelevant. That is, local communities did not differ in species richness, but tended to have species compositions idiosyncratic to each site. Given that cavity‐nesting bees and wasps are highly mobile organisms (e.g., Gathmann & Tscharntke, 2002; Greenleaf et al., 2007; Klein et al., 2004, Guedot et al., 2009) one might expect a more uniform host distribution. However, previous studies in the same study area found high spatial variability in local community composition of bees (both cavity‐ and ground‐nesting) (Torné‐Noguera et al., 2014) and pollinators in general (Reverté et al., 2019). In our study, less than 30% of the variability in host composition can be attributed to foraging distance limitations (as evidenced by the weak correlation between host β‐diversity and geographic distances); the rest of the spatial variability might be attributed to other factors such as food and nesting resource distribution, immigration history, philopatry, and topoclimatic variation (Torné‐Noguera et al., 2014). Information on parasitoid mobility is scarce and controversial (Jeffs & Lewis, 2013), but the general belief is that parasitoids are less mobile than their hosts (Cronin & Reeve, 2005; Jeffs & Lewis, 2013). However, the ability of insects to disperse is usually associated to body size (Shegelski, Evenden, & Sperling, 2019) and, in our study, the body size of most parasitoids is similar to that of their hosts (unpublished data). We can thus assume similar mobility for hosts and parasitoids. Accordingly, we found similarly high spatial variability in parasitoid and in host composition. However, in contrast to hosts, and contrarily to our second hypothesis, parasitoids did not show any distance decay pattern.

Ecological theory predicts a distance decay pattern in community similarity (Nekola & White, 1999; Soininen et al., 2007). Distance decay of species composition of the lower trophic level seems to be widespread (Carstensen et al., 2014; Dáttilo et al., 2013; Novotny, 2009; Trøjelsgaard et al., 2015). On the other hand, composition dissimilarity of the higher trophic level, has been found to either increase (Dáttilo et al., 2013; Trøjelsgaard et al., 2015), decrease (Novotny, 2009) or not vary (Carstensen et al., 2014) with geographical distance. Discrepancies among these studies, including ours, may be due to the fact that these studies have been conducted on different biological systems (from more generalized plant–pollinator to more specialized host–parasite systems), in contrasted habitat types (from rupestrian fields and shrublands to dense rainforests), at different levels of community isolation (inland versus islands), and at different geographical distances (from 600 m to 500 km). Even then, it is surprising that parasitoids and hosts do not follow similar geographic patterns in our study. In relation to this, our results show that the structure of the parasitoid community is highly dependent on the host community, since abundance and richness of the two communities were highly correlated. Since parasitoids tend to have high levels of trophic specialization (Hawkins, 1994; Jeffs & Lewis, 2013; Poulin et al., 2011), we might expect a high correspondence between host and parasitoids communities. Thus, former studies conducted in contrasted habitat types also showed a high dependency of the community structure of the higher trophic level on the lower trophic level (Albrecht et al., 2007; Ebeling, Klein, Weisser, & Tscharntke, 2012; Osorio et al., 2015; Weiner, Werner, Linsenmair, & Blüthgen, 2014). However, and again refuting our second hypothesis, we did not find a strong matching in species composition between hosts and parasitoids across space, indicating that host‐parasitoid systems can be less specialized than previously thought (Blüthgen et al., 2007; Fontaine et al., 2009; Price, 1997; Schowalter, 1996). Our “parasitoid” assemblage included mostly true parasitoids (18 species), but also some cleptoparasites (7), predators (1), and scavengers (1). Although true parasitoids are often thought of as highly specialized two of the most abundant true parasitoids in our study, the chalcid wasp *Melittobia acasta* and the bee‐fly *Anthrax anthrax* had very wide host ranges (13 and 10 host species, respectively, including both bees and wasps). By comparison, the predator *Trichodes leucopsideus* and scavenger *Trogoderma* sp. 1 interacted with 6 and 3 host species, respectively, again including both bees and wasps. At any rate, even in the absence of strong environmental gradients or apparent dispersal barriers, our metacommunity was composed of a mosaic of idiosyncratic local communities at a small spatial scale.

Regarding our second objective, we found that the spatial variability of host‐parasitoid interactions was high (mean β_WN_ = 0.83), and notably higher than that of the host (β_H_ = 0.59) and parasitoid (β_H_ = 0.60) communities. Again, species turnover was the main factor contributing to this variability, which agrees with our third hypothesis. There is a vast body of literature on the causes of variation in species composition in ecological communities; however, less effort has been invested in understanding how interactions between species vary. Interaction dissimilarity is primarily attributable to dissimilarity in species composition, but is also affected by inherent variability even when the two species co‐occur. Such interaction‐inherent variability may be due to neutral effects (interactions are more abundant where populations are more abundant, Canard et al., 2014), competition effects (Novotny, 2009; Poisot et al., 2015) and/or environmental conditions differently filtering not only species but also their interactions (Poisot et al., 2017). For these reasons, higher variability in interaction composition than in species composition is expected. Given the high level of specialization in antagonistic networks (Blüthgen et al., 2007), species turnover would be expected to be the major driver of interaction dissimilarity (Olesen, Stefanescu, & Traveset, 2011), with a lesser contribution of rewiring (shared species interacting differently). Our results (interaction dissimilarity due mostly to species turnover (~75%) rather than rewiring (~25%)) agree with this pattern and with previous studies (Novotny, 2009, Norfolk et al., 2015, Simanonok & Burkle, 2014, Trøjelsgaard et al., 2015; but see Carstensen et al., 2014 for a similar contribution of both components of interaction diversity). These studies, including ours, cover a variety of interaction systems (plant–pollinator, plant–caterpillar, and host‐parasitoid) and a range of geographical scales (from 400 m to 500 km). Thus, species turnover appears to be the main driver of network β‐diversity across biological systems and spatial scales of observation.

In a continuous and homogeneous habitat, we would expect spatial differences in the interaction patterns of species to be explained mostly by neutrality (Canard et al., 2014) and competition, rather than by environmental filtering (Poisot et al., 2017). In relation to this, we found that more abundant host species had higher rates of parasitism. Such a relationship may be explained by two mechanisms. First, prey abundance may influence prey choice, as predators with the ability to prey on different hosts obtain greater returns by learning how to handle the most abundant prey and focusing on it (Ishii & Shimada, 2012). Second, specialized antagonists, with a restricted range of potential preys/hosts, may not be able to build stable populations on locally rare hosts (Newman, 2002). Therefore, our metacommunity would correspond to systems in which focusing on the most abundant resource provides an insurance against situations of strong species turnover (Canard et al., 2014; Gravel, Massol, Canard, Mouillot, & Mouquet, 2011). On the other hand, overall parasitism rate was not explained by overall host abundance at the plot level. Other studies on cavity‐nesting bees and wasps have found similar results (Albrecht et al., 2007; Tylianakis, Tscharntke, & Klein, 2006), but others have found a positive correlation between parasitism rate and host abundance (Steffan‐Dewenter, 2003).

In relation to our third objective, interaction similarity between local networks and their counterparts in the regional network was high, as reflected by the low β’_OS_ (mean ± *SD *= 0.17 ± 0.1). In other words, interactions in local networks were highly nested in the regional network, and when partners co‐occurred locally, interactions were usually realized. Interaction nestedness in our system resulted from a combination of (a) species interacting similarly across plots, and (b) large differences between plots in species composition. Because local communities were highly idiosyncratic, the contribution of each plot to the regional web was high. In a mammal–ectoparasite regional network, Poisot et al. (2012) also found interactions to be highly idiosyncratic at the local level. However, the mechanisms explaining their result were completely different. As opposed to our study, they obtained high values of β’_OS_ indicating that many interactions only occurred in a reduced number of local webs. They conclude that interactions are highly determined by local conditions (high interaction filtering). Conversely, in our study the proportion of shared species between local webs is low (high levels of community dissimilarity), and shared species interact similarly (very low levels of rewiring). As a consequence, even if many interactions only occur in a few local webs, β’_OS_ values are low. In other words, in our community interactions are site‐idiosyncratic not because interaction composition per se is determined by local conditions, but because species composition is determined by local conditions. These results agree with a previous study in the same area in which composition of the bee community was found to be conditioned by local effects (Torné‐Noguera et al., 2014). Our results are also congruent with a study on cavity‐nesting bees and wasps that found local factors to be much more important than landscape factors in determining community and network structure and composition (Osorio et al., 2015).

As with the parasitoid community, interaction dissimilarity did not increase with geographic distance. So far, few studies have explored interaction similarity decay and they do not provide consistent results. Some works found distance decay patterns (Carstensen et al., 2014, Trøjelsgaard et al., 2015, Dáttilo & Vasconcelos, 2019), but others did not (Dáttilo et al., 2013; Simanonok & Burkle, 2014). Although the low number of studies prevents the establishment of robust conclusions, the studies that did find interaction similarity decay patterns were performed at much larger spatial scales that the ones that did not.

Our fourth objective was to establish whether interaction spatial variability was better explained by the spatial variability of the host or the parasitoid communities. Our predictive models show that both host and parasitoid beta‐diversity are important. However, parasitoids (or parasitized hosts) explain a much greater fraction of the variability, which contradicts our fourth hypothesis that interaction β‐diversity will be better predicted by host β‐diversity than by parasitoid β‐diversity. Our results are counter to various studies finding a greater contribution of the lower trophic level (Carstensen et al., 2014; Norfolk et al., 2015; Novotny, 2009; Simanonok & Burkle, 2014). However, these studies reported greater turnover of the lower trophic level, whereas in our study the host and parasitoid communities displayed similar levels of turnover. In addition, hosts and parasitoids in our study appear to be differently affected by geographic distance. Our results imply that there is no easy short‐cut to characterize the spatial distribution of host‐parasitoid interactions. Even at the small spatial scale used in our study, a large fraction of the interaction variability is missed if only hosts (or parasitoids) are sampled.

Overall, our study demonstrates that communities of mobile organisms and their interactions show a high level of variability even at local scale (~500 m) in a homogeneous habitat. Parasitoid spatial turnover is the major driver of the observed spatial heterogeneity in host‐parasitoid interactions. Nonetheless, interactions are also subjected to their own intrinsic variability. Thus, it is important to take into account both levels of variability (communities and interactions) to adequately characterize ecological function. Our results also show the need to sample a large number of plots to adequately characterize a regional network even across a continuous and uniform habitat. Distinguishing between the two components of interaction β‐diversity is essential to understand the drivers of such diversity. When interaction β‐diversity is mostly due to interaction rewiring, the resulting metaweb will reflect the regional, but not the local behavior of interacting species. In contrast, when interaction β‐diversity is mostly due to species turnover, the metaweb will reflect the local behavior of interacting species (Poisot et al., 2012). Our system falls within the latter scenario, with high levels of species turnover and low levels of rewiring. However, even then it is necessary to sample a sufficiently large number of plots because species composition is highly dependent on local factors and subjected to variation at very small scales. Our results have important implications for conservation policy. Even within a homogeneous landscape it is necessary to preserve large areas of land to cover the entirety of the regional taxonomic diversity along with all potential interactions and ecological functions.

## Supporting information

Appendix S1
Appendix S2
Appendix S3

## Data Availability

Host‐parasitoid interactions data: Dryad https://doi.org/10.5061/dryad.kprr4xh1t.
