# Supplementary material for: Spatial variability of hosts, parasitoids and their interactions across a homogeneous landscape
Source: Ecol Evol. 2020 Mar 4;10(8):3696–705. doi: 10.1002/ece3.6158 (PMC7160165; doi:10.1002/ece3.6158)

**Supporting information. Spatial variability of hosts, parasitoids and their interactions across a homogeneous landscape**

**APPENDIX A**

**Figure A1.** Map showing the distribution of plots used in this study.

**
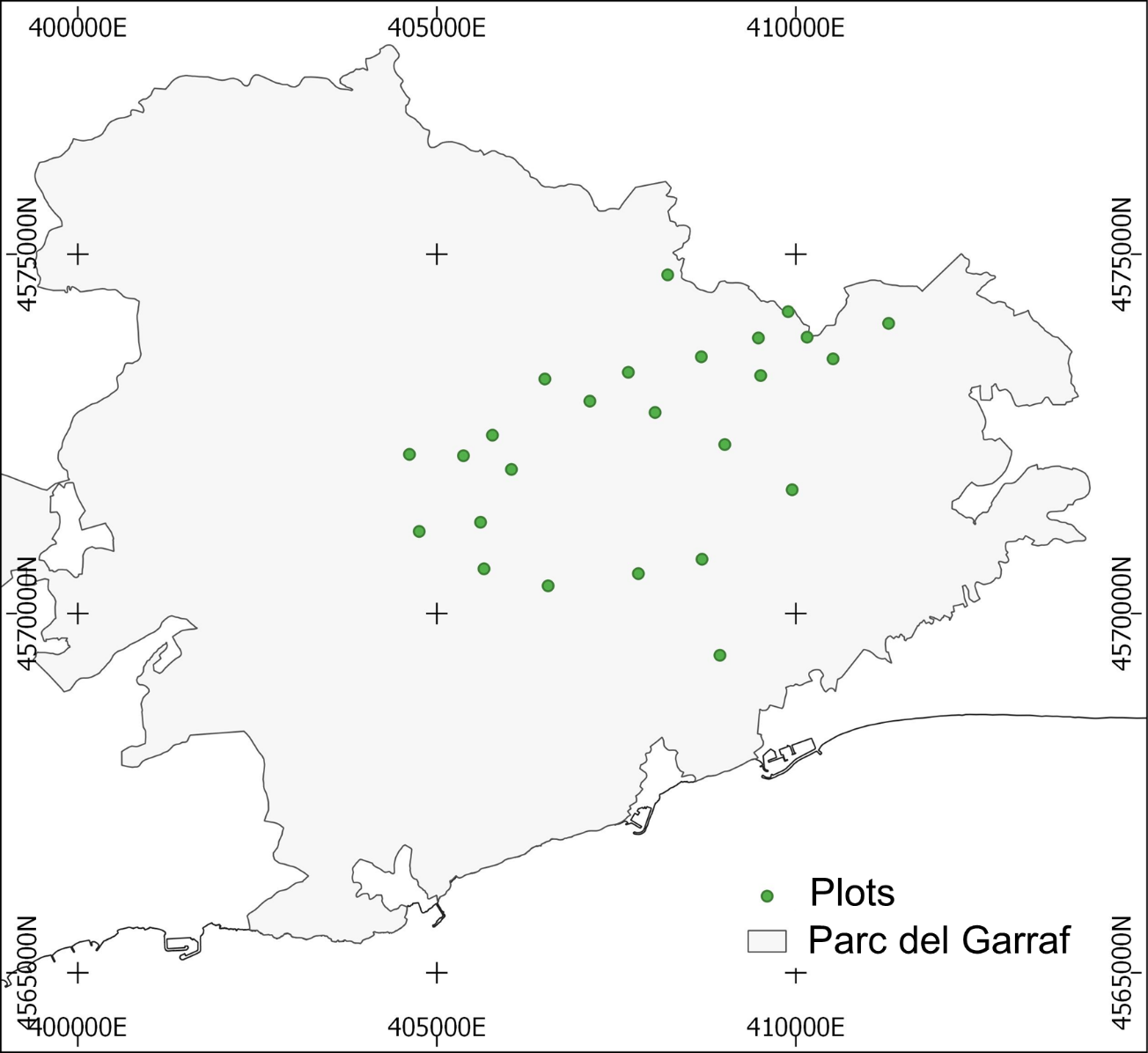
**

**APPENDIX B**

**Table B1.** Cavity-nesting bee and wasp species (hosts) and their codes.

| **Code** | **Species** | **Bee/wasp** |
| --- | --- | --- |
| H01 | *Ampulex ruficollis* | Wasp |
| H02 | *Ancistrocerus longispinosus* | Wasp |
| H03 | *Ancistrocerus sp.1* | Wasp |
| H04 | *Anthidium florentinum* | Bee |
| H05 | *Anthidium nigricolle* | Bee |
| H06 | *Auplopus carbonarius* | Wasp |
| H07 | *Chelostoma edentulum* | Bee |
| H08 | *Diodontus sp.1* | Wasp |
| H09 | *Dipogon sp.1* | Wasp |
| H10 | *Dipogon sp.2* | Wasp |
| H11 | *Dipogon sp.3* | Wasp |
| H12 | *Euodynerus sp.1* | Wasp |
| H13 | *Euodynerus sp.2* | Wasp |
| H14 | *Heriades crenulatus* | Bee |
| H15 | *Hoplitis adunca* | Bee |
| H16 | *Hylaeus hyalinatus* | Bee |
| H17 | *Hylaeus taeniolatus* | Bee |
| H18 | *Isodontia mexicana* | Wasp |
| H19 | *Megachile apicalis* | Bee |
| H20 | *Megachile ericetorum* | Bee |
| H21 | *Megachile rotundata* | Bee |
| H22 | *Nitela fallax* | Wasp |
| H23 | *Nitela sp.1* | Wasp |
| H24 | *Nitela truncata* | Wasp |
| H25 | *Osmia bicornis* | Bee |
| H26 | *Osmia caerulescens* | Bee |
| H27 | *Osmia latreillei* | Bee |
| H28 | *Osmia melanogaster* | Bee |
| H29 | *Osmia nasoproducta* | Bee |
| H30 | *Osmia submicans* | Bee |
| H31 | *Osmia tricornis* | Bee |
| H32 | *Passaloecus gracilis* | Wasp |
| H33 | *Passaloecus pictus* | Wasp |
| H34 | *Pison atrum* | Wasp |
| H35 | Pompilidae *Gen. sp.1* | Wasp |
| H36 | *Psenulus fuscipennis* | Wasp |
| H37 | *Solierella compedita* | Wasp |
| H38 | *Solierella sp.1* | Wasp |
| H39 | *Trypoxylon sp.1* | Wasp |
| H40 | *Trypoxylon sp.2* | Wasp |

**Table B2.** Nest associate species (parasitoids, cleptoparasites, predators and scavenger) and their codes.

| **Code** | **Species** | **Order** | **Interaction type** |
| --- | --- | --- | --- |
| P01 | *Anthrax anthrax* | Diptera | Parasitoid |
| P02 | *Anthrax sp.2* | Diptera | Parasitoid |
| P03 | *Calliphoridae sp.1* | Diptera | Parasitoid |
| P04 | *Chaetodactylus osmiae* | Sarcoptiforme | Cleptoparasite |
| P05 | Chalcididae *Gen.* sp*.1* | Hymenoptera | Parasitoid |
| P06 | *Chrysis ignita* | Hymenoptera | Parasitoid |
| P07 | *Chrysura sp.1* | Hymenoptera | Parasitoid |
| P08 | *Coelioxys echinata* | Hymenoptera | Cleptoparasite |
| P09 | *Cystomutilla sp.1* | Hymenoptera | Parasitoid |
| P10 | *Gasteruption sp.1* | Hymenoptera | Cleptoparasite |
| P11 | *Hedycridium sp.1* | Hymenoptera | Parasitoid |
| P12 | *Leucospis dorsigera* | Hymenoptera | Parasitoid |
| P13 | *Melittobia acasta* | Hymenoptera | Parasitoid |
| P14 | *Zonitis immaculata* | Coleoptera | Cleptoparasite |
| P15 | *Miltogramma sp. 1* | Diptera | Cleptoparasite |
| P16 | *Miltogramma sp. 2* | Diptera | Cleptoparasite |
| P17 | *Omalus sp.1* | Hymenoptera | Parasitoid |
| P18 | *Omalus sp.2* | Hymenoptera | Parasitoid |
| P19 | *Perithous sp.1* | Hymenoptera | Parasitoid |
| P20 | *Physetopoda sp.1* | Hymenoptera | Parasitoid |
| P21 | Pteromalidae *Gen. sp.1* | Hymenoptera | Parasitoid |
| P22 | *Sapyga quinquepunctata* | Hymenoptera | Cleptoparasite |
| P23 | *Toxophora fasciculata* | Diptera | Parasitoid |
| P24 | *Trichodes leucopsideus* | Coleoptera | Predator |
| P25 | *Trichrysis cyanea* | Hymenoptera | Parasitoid |
| P26 | *Trogoderma sp.1* | Coleoptera | Scavenger |
| P27 | *Xorides sp.1* | Hymenoptera | Parasitoid |

**APPENDIX C.**

**Figure C1.** Sample-based host species accumulation curve obtained from 1000 sequences of random site additions. Dotted lines represent the 95% confidence intervals.


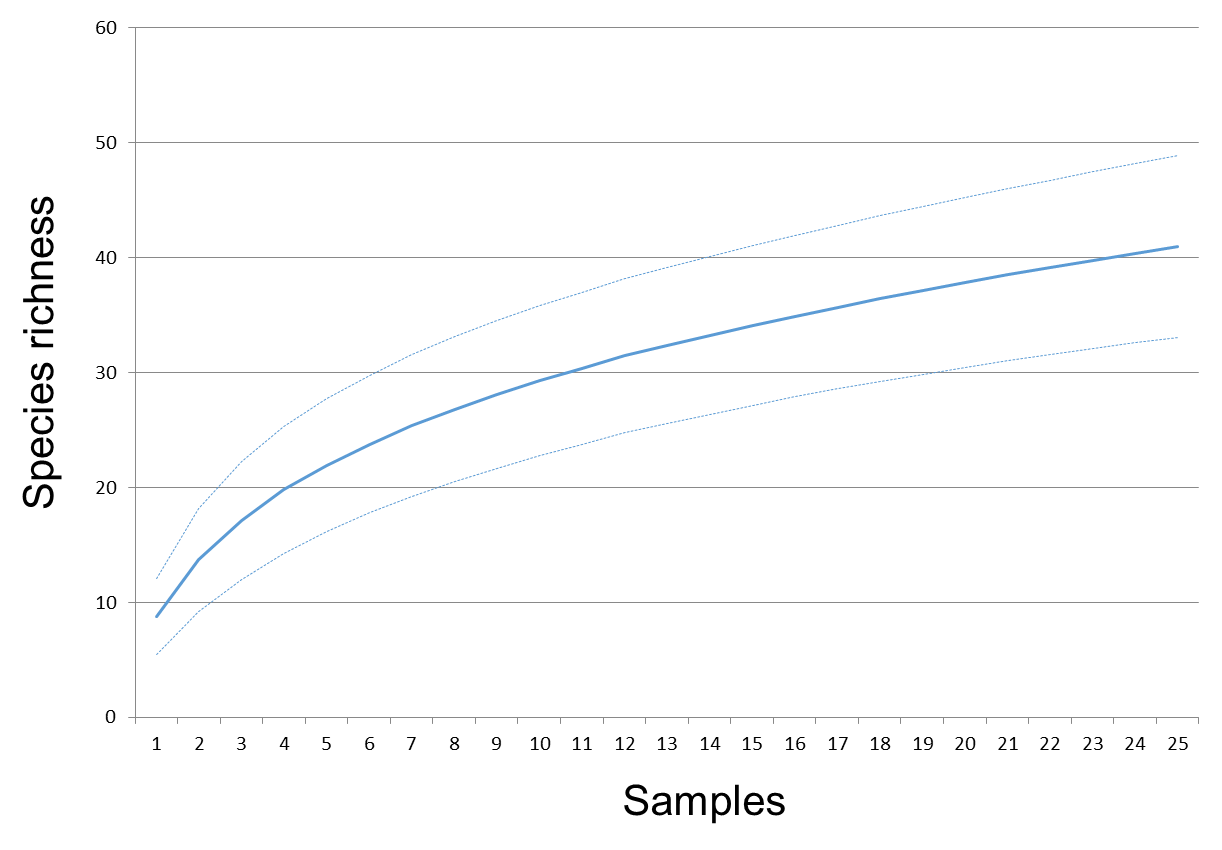

Supplement: Supplementary file 1 — Appendix S1 Appendix S2 Appendix S3 [file ECE3-10-3696-s001.docx]
